# Diseases Transmitted by the Black-Legged Ticks in the United States: A Comprehensive Review of the Literature

**DOI:** 10.7759/cureus.17526

**Published:** 2021-08-28

**Authors:** Juan P Sosa, Maria M Ferreira Caceres, Kuchalambal Agadi, Krunal Pandav, Meghana Mehendale, Jayati M Mehta, Camille Celeste Go, Wanessa Figueiredo Matos, Prathima Guntipalli, Marie-Pierre E Belizaire

**Affiliations:** 1 Division of Research & Academic Affairs, Larkin Community Hospital, South Miami, USA; 2 Family Medicine, Larkin Hospital Palm Springs Campus, Hialeah, USA

**Keywords:** black-legged ticks, ixodes scapularis tick, ixodes scapularis, disease, control

## Abstract

The black-legged tick is endemic to the midwestern, northeastern, western, south-eastern, and southern regions of the United States. There has been an increased burden of black-legged ticks in humans in recent years. COVID-19 pandemic has further heightened this burden. We thereby reviewed the literature to discuss the seasonality, infections, and clinical spectrum of diseases transmitted by the black-legged ticks. We also discuss the reported delay in the diagnosis of these diseases during the pandemic situation, the alpha-gal syndrome, the importance of prompt diagnosis, and early medical intervention with an aim to increase awareness of the black-legged tick-borne diseases.

## Introduction and background

The black-legged tick, also known as *Ixodes scapularis* or *I. scapularis*, is endemic to the United States, Midwest, northeast, west, southeast, and southern regions [1]. Lyme disease is the most common tick-borne disease transmitted by *I. scapularis* in the United States [2]. Each year about 3,00,000 cases of Lyme disease are diagnosed in the United States. The causative agent for Lyme disease is *Borrelia burgdorferi* [3]. Black-legged ticks are associated with various microorganisms including bacteria, protozoan parasites, and viruses [4,5], summarized in Table 1.

**Table 1:** Microorganisms and diseases associated with black-legged ticks

No.	Name of Microorganism	Type of Microorganism	Disease
1	Anaplasma phagocytophilum	Bacteria	Human granulocytic anaplasmosis
2	Babesia microti	Protozoan parasite	Babesiosis
3	Borrelia burgdorferi	Bacteria (Spirochete)	Lyme disease
4	Borrelia miyamotoi	Bacteria (Spirochete)	*Borrelia miyamotoi *disease
5	Bourbon virus	Virus	Bourbon virus disease
6	Powassan virus	Virus	Powassan disease/viral encephalitis
7	Ehrlichia muris eauclarensis	Bacteria	Ehrlichiosis
8	Borrelia mayonii	Bacteria	*Borrelia mayonii* disease

The Center for Diseases Control and Prevention (CDC) suggests knowing about the ticks’ habitat, i.e., grassy, bushy, or wooded areas, or even on animals; treating the clothing and gear with 0.5% permethrin; using Environmental Protection Agency (EPA)-registered insect repellents; and avoiding wooded and bushy areas with high grass and leaf litter [6]. Due to tick-borne infections, increased economic burden from a societal perspective was seen before the COVID-19 pandemic [7,8]. Lyme disease could have an estimated burden of $712 million to $1.3 billion a year on the US healthcare system [9]. The economic burden due to Lyme disease in Maryland in 1997-2000 was approximately $10,000 per case of Lyme disease, including both direct/indirect medical costs and losses in productivity [8]. These costs are influenced by the severity of the disease and stage of diagnosis [10]. Additionally, the COVID-19 pandemic has further heightened the burden due to tick-borne infections, in terms of delay in diagnosis, superadded COVID-19 infection [11,12]. Due to the high burden, research is needed to guide and develop healthcare policy [7]. Moreover, researchers demonstrated that the theory-based tick-borne disease prevention program led to the adoption of precautionary measures by people causing a decreased incidence of tick-borne infections by 60% [13].

Following this, we present a comprehensive review of the diseases caused by the black-legged ticks in the United States. We also discuss alpha-gal syndrome and the effect of COVID-19 on tick-borne infections in the United States.

## Review

1. Brief overview of the life cycle of *Ixodes scapularis*

*I. scapularis* belongs to the Ixodidae family, and it takes multiple molts for this tick to reach adulthood. The tick survives for about two to four years [1,14,15].* I. scapularis* depends on three natural hosts for its blood meal; it is crucial to complete its life cycle, including the larval stage, nymphal stage, and adult [1,14,15]. The larva and the nymph have a rodent, *Peromyscus leucopus*, as their primary host, and a white-tailed deer, *Odocoileus virginianus*, serves as the host for the mature ticks (adult stage) [1]. Multiple pathogens can be acquired, multiplied, and transmitted due to the midgut and the tick's salivary glands during the blood meal [14-16]. However, the transmission of the *Powassan virus* and *Borrelia Miyamotoi* is known to occur transovarially as well. Transmission of pathogens to humans, carried by *I. scapularis*, mainly occurs during the nymphal stage [14]. The nymphs molt into mature ticks in their second year during autumn [14]. Female adult ticks feed on the white-tailed deer, whereas male adult ticks do not require a blood meal to survive. Male ticks die after mating, and the female ticks are known to lay eggs in thousands during May [14].

2. Black-legged tick-borne Infections in the United States

2.1. Anaplasma phagocytophilum

*Anaplasma phagocytophilum* belongs to the *Rickettsiale *order. It is an obligate intracellular gram-negative bacteria [17,18]. It causes anaplasmosis, also known as human granulocytic anaplasmosis (HGA) [19].

The CDC had reported an increased number of anaplasmosis cases from 348 in 2000 to 5,762 in 2017. On the other hand, in 2018, 4008 cases of anaplasmosis were reported. The majority of the patients registered were males above 40 years of age. The case fatality rate was persistently low (<1%) [20]. Anaplasmosis and ehrlichiosis are the second-most common tick-borne diseases reported in 2019, after Lyme disease [20]. Eight states, including Vermont, Maine, Rhode Island, Minnesota, Massachusetts, Wisconsin, New Hampshire, and New York, account for around nine in 10 of all the reported cases of anaplasmosis [20].

Anaplasmosis usually presents non-specific symptoms such as fever, chills, malaise, myalgias, headache, and rarely a rash. There are also reports of non-specific gastrointestinal (G.I.) or respiratory symptoms [19]. Immunocompromised people might be at an increased risk of hospitalization and severe outcomes [19,20]. In addition, co-infection with other tick-borne organisms (Anaplasma, Lyme, and Babesia) may occur due to *I. scapularis* being the common vector [19].

Culture, histopathology, polymerase chain reaction (PCR), or serology is used to diagnose the disease. Intracytoplasmic aggregates of Anaplasma in peripheral blood neutrophils can be detected in 20%-80% of symptomatic patients. In infected patients, lymphoid organs (bone marrow, liver, spleen, and lymph nodes) show changes in mononuclear phagocytes. Lungs may also be affected due to the systemic inflammatory response. Periportal lymphohistiocytic infiltrates, focal splenic necrosis, mild interstitial pneumonitis, and pulmonary hemorrhage are also seen in severe cases with organ damage [19]. The treatment is detailed in Table 2.

**Table 2:** Treatment of black-legged ticks CNS, Central nervous system; POWV, *Powassan virus.*

No.	Pathogen	First-Line Treatment	Additional Information
1	Anaplasma phagocytophilum	Doxycycline 100 mg administered twice daily for 14 to 21 days; OR at least three days after defervescence. If co-infection with Lyme, give treatment for extra 10 days.	Potential adverse effects of doxycycline: life-threatening allergies and tooth discoloration in children < 8 years old.
2	Babesia microti	Atovaquone: 750 mg orally twice a day + azithromycin: first day: 500–1000 mg orally; subsequent days: 250–1000 mg per day OR clindamycin: 600 mg orally three times a day, or 300–600 mg IV every 6 hours + quinine: 650 mg orally every 8 hours. In addition to these, some patients, including severely ill patients, might need additional supportive care.	Quinine and clindamycin can be safely used to treat symptomatic pregnant women. Atovaquone can be administered with care in lactating women feeding infants who weigh <5 kg. Atovaquone is considered to be safe to be used in children who weigh >5 kg. Weighing the benefits and risks, azithromycin can be administered to pregnant and lactating women with caution. From 6 months to 16 years of age, azithromycin is considered a safe drug to treat Babesiosis. Benzyl alcohol is present in the parenteral form of clindamycin, which is known to cause “Gasping Syndrome” in premature infants.
3	Borrelia burgdorferi	If >8 years old with early, localized sickness: doxycycline 100 mg orally twice daily OR doxycycline 200 mg once daily for 10 days. Patient < 8 years old: amoxicillin 500 mg orally three times daily for 14 days OR cefuroxime orally three times daily for 14 days. If the person is allergic or intolerant to doxycycline, amoxicillin, and cefuroxime, they can be given macrolides: azithromycin, clarithromycin, or erythromycin. However, these drugs are of lower efficacy. People undergoing macrolides treatment should be monitored. Pregnancy: ceftriaxone. Carditis or CNS involvement: ceftriaxone OR doxycycline. The ocular feature of zoonotic disease: topical steroids + IV cephalosporin.	Post-treatment, Lyme disease syndrome is experienced by some patients. Antibiotics do not resolve these symptoms.
4	Bourbon virus	No medications; only symptomatic treatment.	-
5	Powassan virus	No specific medications. Severe disease, supportive treatment with respiratory support, intravenous fluid steps to reduce cerebral edema. POWV neuroinvasive disease: treated with high-dose corticosteroids. POWV encephalitis treated with IVIg.	-
6	Ehrlichia muris eauclarensis	Doxycycline 5-10 mg/kg every 12 hours for 5-7 days.	-
7	Borrelia mayonii	Doxycycline 100 mg orally twice daily or 200 mg once daily for 10 days.	-
8	Borrelia miyamotoi	Tick-borne relapsing fever (TBRF): Adults: a 7-day course of oral or parenteral of chloramphenicol (500 mg) 6 hourly daily for 7 days OR doxycycline (100 mg) twice daily for 7 days OR erythromycin (500 mg) 6 hourly daily for 7 days OR tetracycline (500 mg oral/250 mg parental) 6 hourly daily for 7 days OR parenteral penicillin G 60,000 IU daily for 7 days. Children < 8 years or pregnant women: penicillin G or erythromycin.	Jarisch–Herxheimer reaction with hypotension, tachycardia, chills, rigors, diaphoresis, and marked elevation of body temperature. Partial agonist meptazinol seems to reduce the severity of these symptoms due to Jarisch–Herxheimer's reaction; death is also reported as this reaction’s complication secondary to cardiovascular collapse.

2.2. *Babesia microti*

Babesiosis is caused by a protozoan (Piroplasmida: Babesiidae) that infects farm animals and causes zoonotic infection in humans [21]. Though there are more than 100 Babesia species, *Babesia microti* is the most common type, and it is transmitted by *I. scapularis*. In 2018, the CDC reported 2,161 cases of Babesiosis in the United States [22]. In 2019, 4.75% of tick-borne infections were caused by *B. microti* [22].

Babesiosis can be acquired through the bite of an infected tick or by receiving a blood transfusion from an asymptomatic infected donor or rarely through vertical transmission [22]. Tonnetti et al. conducted a clinical trial with blood donation samples from Massachusetts, Connecticut, Minnesota, and Wisconsin. They screened (by immunoassay and PCR) 5,06,540 donations from June 2012 to May 2018 for *B. microti*, of which 1299 were positive. They propose that by deploying routine screening of *B. microti* in endemic areas, transfusion-transmitted Babesiosis can be mitigated [23]. The clinical manifestations of the disease are due to the multiplication of the blood-stage parasites, and that can even cause lysis of the erythrocytes [24].

Most of the individuals affected by Babesiosis appear to be asymptomatic. However, a group of elderly and immunocompromised patients, including asplenic patients, are at increased risk of developing adverse clinical complications such as hemolysis, thrombocytopenia, acute respiratory distress, renal failure, hepatic compromise, disseminated intravascular coagulation (DIC), altered mental status, and multiorgan failure ultimately causing death [21,25]. Some patients might require supportive care, such as antipyretics, vasopressors, blood transfusions, exchange transfusions, mechanical ventilation, or dialysis [26]. However, asymptomatic patients usually do not need to be treated [26]. The combination of drug therapy is detailed in Table 2.

2.3. *Borrelia burgdorferi*

*Borrelia burgdorferi* is a spirochete, transmitted by *I. scapularis*. It is the causative agent of a zoonotic infection commonly known as Lyme disease [27]. Lyme disease burden is high. In 2018, the estimated cost for acute disease treatment was $4.8 billion and for chronic disease was $9.6 billion in the United States [28]. In the northeastern and midwestern United States, it is mainly transmitted by *I. scapularis* and in the western states by *Ixodes pacificus* [29]. Clinically, Lyme disease manifests in three stages seen at different intervals from the tick bite [29]. Within days to weeks of a tick bite, 70%-80% of patients present with a “bull’s eye rash,” also known as “target rash” or erythema migrans; a diagnostic feature of the disease [29,30] is commonly seen on the thighs, groin, and the axilla [31].

The early or disseminated phase occurs after few weeks to months, characterized by fever, headache, migratory arthralgias, myalgias, headache, fatigue, neurological (20%), and cardiac manifestations in some cases [2,29,32]. Lymphocytic meningitis, facial nerve palsy (5%), and sensory or motor radiculoneuritis are commonly seen neurological features in the United States [2,29]. Cardiac complications may occur in around 0.3%-10% of Lyme disease patients and include atrioventricular blocks of varying degrees, myopericarditis, or cardiomegaly [31].

After months to years, Lyme arthritis, commonly involving the knee joint, is seen in stage 3 or Late Lyme disease, especially in people who do not receive treatment in the early stage of the disease [2,29,32]. Lyme disease can be diagnosed either clinically or by laboratory methods. The latter are of two types, direct or indirect methods. Direct diagnostic tests include PCR assays [33,34]. Indirect tests include enzyme-linked immunosorbent assays (ELISA) and western blot [33]. CDC recommends a two-step testing process (positive ELISA followed by western blot) to detect Lyme disease [35]. However, recently, CDC has suggested that a consecutive ELISA test can be used as an alternative to the western blot test [36]. The treatment [2,34,37] is detailed in Table 2.

2.4. Bourbon virus

*Bourbon virus* (BRBV) was identified first in a patient with fever and a history of tick bite in Bourbon County, Kansas City, United States [38]. Subsequently, other human BRBV infections were reported from the midwestern and southern United States [39]. BRBV belongs to the *Orthomyxoviridae *family and *Thogotovirus *genus worldwide [38,40]. The genus *Thogotovirus *has a negative-sense RNA genome of six segments. *Thogotovirus *is transmitted by both hard or soft ticks [41].* I. scapularis* and Lone star tick, *Amblyomma americanum*, are associated with BRBV transmission in humans and other vertebrates [42,43].

BRBV prevalence in potential vectors remains unclear; additional field studies are required. However, the very low infectious nymph prevalence and varied male adult rates show that other transmission cycles for BRBV might be possible [39]. Patients with impaired innate immunity might be at the risk of severe BRBV infection and benefit from an antiviral treatment like ribavirin and favipiravir [44]. BRBV infections have been identified with a history of tick bites associated with fever, rash, headache, malaise, nausea, vomiting, thrombocytopenia, and leukopenia [40]. The treatment [45] is detailed in Table 2.

2.5. Powassan virus

*Powassan virus* (POWV), a member of the tick-borne encephalitis serocomplex of flavivirus, causes Powassan disease [46]. It was first identified in a young boy in 1958 who died with encephalitis in Powassan, Ontario, Canada [47]. The genome of POWV includes 11 kb of single-stranded, positive-sense RNA. There is only limited data available regarding the various strains and genomic studies on POWV [48-50]. However, POWV replication and its causation in humans are known by research on the closely associated tick-borne encephalitis virus (TBEV) [51]. The TBEV genome and the POWV genome consist of single-stranded, positive-sense RNA [52]. POWV has two distinct genotypes that are serologically difficult, and both are transmitted through ticks: POW lineage 1 (POW-L1) and deer tick virus or POWV lineage 2 (DTV). However, in POWV, lineage I is maintained by *Ixodes cookei*, also known as the Groundhog ticks, whereas DTV lineage II is maintained by *I. scapularis* [49].

POWV causes rare severe neuroinvasive diseases in humans. The incubation period ranges between one and five weeks. The prodromal phase includes headache, sore throat, drowsiness, and disorientation, followed by severe neurological involvement with encephalitis, meningoencephalitis, and aseptic meningitis. The encephalitis phase includes prolonged fever, vomiting, speech difficulties, loss of coordination, seizures, and paralysis [51]. Over 50% of survivors from encephalitis might end up with several long-lasting neurological sequelae, including memory problems, hemiplegia, muscle wasting, and severe headache [49]. Diagnosis of POWV during the early viremic phase of the disease is made by detecting specific nucleic acid or viral antigen and by viral isolation. In later stages of the disease, detection of POWV-specific IgM and IgG antibodies by serological testing is diagnostic [51]. Most of the studies show non-specific magnetic resonance imaging (MRI) abnormalities for POWV cases. However, certain studies show that MRI in POWV patients shows T2/fluid attenuation inversion recovery (FLAIR) hyperintensities within the brainstem and extensions into deep gray structures and cortex [49]. POWV and DTV are usually serologically indistinguishable, but recent studies show that PCR primers specific to the envelope-coding region (NS5 coding region) can be used to differentiate them [53]. The treatment [49,53-55] is detailed in Table 2.

2.6. *Ehrlichia muris eauclarensis*

Ehrlichia species is an obligate intracellular bacteria transmitted by hard ticks. Most of the Ehrlichia are transmitted by the *Amblyomma americanum* tick, prevalent in southeast, south-central, and mid-Atlantic states. Nevertheless, the *Ehrlichia muris*-like (EML) genus of Ehrlichia is transmitted by *I. scapularis*, especially in Wisconsin and Minnesota [13].

In an epidemiologic study of EML in 69 patients between 2004 and 2013, it was found that symptoms due to EML are more common in immunocompromised patients [56]. Sixty percentage of children and 30% of adults with Ehrlichiosis present with skin rash [57]. Ehrlichiosis presents with symptoms such as fever, malaise, headache, and myalgia. The common hematologic picture in them are anemia, thrombocytopenia, lymphopenia, leukopenia, and increased aspartate aminotransferase and alanine aminotransferase enzymes. They also noted that two patients were co-infected with *B. burgdorferi* [56]. The rare complications occurring in patients with Ehrlichiosis are renal failure, acute respiratory distress syndrome, neurologic disorders, and DIC [58]. Ehrlichiosis is diagnosed clinically based on signs and symptoms, history of a tick bite, and exposure to the endemic areas and confirmed by laboratory tests such as PCR, indirect immunofluorescence antibody (IFA) assay for immunoglobulin G (IgG), culture isolation, and immunohistochemical assays. However, treatment must be initiated promptly without waiting for the lab reports [57]. The treatment [57] is detailed in Table 2.

2.7. Borrelia mayonii

*Borrelia mayonii *species nova has recently been reported as a unique spirochete inflicting Lyme disease in North America [59]. The morphology matches that of antecedently delineated species of the genus Borrelia. *B. mayonii *is often distinguished from all different Lyme Borreliosis-group spirochetes by multi-locus sequence analysis of eight housework loci [60]. Patients infected with *B. mayonii *present with either focal or diffuse rash, nausea, vomiting, along with neurological symptoms in some cases [59,60]. PCR shows a high spirochete load in the blood during the acute phase of the infection [60]. The treatment [4] is detailed in Table 2.

2.8. Borrelia miyamotoi

*Borrelia miyamotoi* (*B. miyamotoi*) is known to be one of the causative agents of *B. miyamotoi* disease (BMD), also known as hard tick-borne relapsing fever (TBRF) [61] transmitted by *I. scapularis* in the United States [62]. Additionally, in a study done by Jobe et al. (2016), seven cases of typical relapsing fever caused by *B. miyamotoi* were confirmed in Wisconsin [63]. BMD is characterized by episodes of high fever, nausea arthralgia, myalgia, nausea, arthralgia, myalgia, and fatigue, which can last for several weeks post-antibiotic therapy [64,65]. Monocytosis and thrombocytopenia are classically seen in American BMD [65]. In addition, elderly patients and immunocompromised patients can present with neurological complications such as meningoencephalitis [64]. The treatment [66,67] is detailed in Table 2.

3. Alpha-gal syndrome

Alpha-gal syndrome (AGS) is an IgE antibody-mediated allergic reaction to galactose sugar at alpha 1,3 position [68]. Typically, patients who have tolerated mammalian meat for many years suddenly develop an allergic reaction after being sensitized by a tick bite. Alpha-galactose sugar is found in the tick’s saliva [69,70]. Though bite by the Lone Star ticks (*A. americanu*m) is the most common cause of sensitizer for the alpha-gal allergic reaction [71], black-legged ticks are also known to precipitate this condition [72,73]. On exposure to mammalian meat, or products (medications, cosmetics, gelatin, etc.), sensitized patients experience allergic symptoms (from mild to life-threatening). These symptoms range from pruritus, rash, nausea, vomiting, and dizziness to breathing difficulty and hypotensive shock [74]. The management for AGS is the same as that for any other allergic reaction, with epinephrine, antihistamines, and in case of shock with vasopressors and fluid. AGS can be prevented by preventing tick bites [74], and sensitized individuals should avoid foods and products, which contain alpha-gal [75].

4. Public health strategies for prevention and treatment of tick-borne disease

Various public health strategies recommended against tick-borne infections are effective educational interventions for promoting behavioral modification. These include promoting the use of repellents, wearing protective clothing while in endemic areas, checking for ticks, and removing them at the earliest. The definitively preventive measure of vaccination against tick-borne infections is also under trial [76,77].

5. Current significance of tick-borne infections amid the ongoing pandemic situation

Due to the COVID-19 pandemic, either misdiagnosis or delayed diagnosis of tick-transmitted infection has been reported. This was mainly because of non-specific, overlapping early symptoms. The overlapping symptoms were febrile illnesses, fatigue, or body aches [78]. Wormser et al. reported that patients were initially tested for COVID-19; once ruled out, they were subsequently tested for other pathogens. Due to the delay in diagnosis and treatment, the patient developed cardiac complications from Lyme disease. Skin rash was missed because the initial consultation was a teleconsultation [11]. Tick exposure season was ongoing in the spring of 2020 at the onset of the pandemic. Since screening for COVID-19 symptoms was not yet specific, late diagnosis of Lyme disease led to ocular palsy due to disseminating infection [78].

Jha et al. explored significant complications that could arise in the late or misdiagnosis of Lyme disease. One major complication, although uncommon, is optic neuritis that was observed in a patient infected with *B. burgdorferi*. Other complications included joint involvement, meningitis, skin lesions, carditis, and cranial nerve involvement. To treat neurological complications of Lyme disease, ceftriaxone, cefotaxime, and penicillin G are indicated. Another highly effective antibiotic is oral doxycycline. Early diagnosis is crucial to prevent irreversible complications [79]. It is recommended that in high endemic areas or patients with a known history of tick bites, appropriate testing should be conducted simultaneously as COVID-19 tests to prevent delayed diagnosis leading to complications. Rose et al. has reported patients who tested positive for COVID-19 and Lyme disease. The Global Lyme Alliance has reported cases of COVID-19 in patients with chronic tick-borne co-infections with Lyme, Ehrlichia, and Babesia. They mention that due to immunosuppressive medications such as hydroxychloroquine, which these patients are prescribed, there is an increased chance of false-negative COVID-19 tests [80]. So far, they have not recorded any increased severity of symptoms or risk of complications due to COVID-19 in patients with pre-existing chronic tick-borne infections. Limited data is available regarding COVID-19 mRNA vaccination in patients with chronic tick-borne infections like Lyme disease [81].

## Conclusions

The tick-borne infections cause a social and economic burden in the United States, and a co-infection of various bacteria, viruses, and protozoa is common with the tick bite. Ongoing pandemic situation further aggravated this by creating a diagnostic dilemma due to overlapping of symptoms or co-infection or superadded infection, non-conclusive tests, and increased transfer of primary care to the telehealth version. Active public health promotion measures must be taken in the endemic areas to encourage preventive measures against tick-borne infections. Prospective cohort studies in the endemic areas are needed to help reverse the increasing number of cases.
